# *Rahnella aquatilis* Isolated from *Aedes albopictus* Impairs Mosquito Reproduction Capacity

**DOI:** 10.3390/insects16030257

**Published:** 2025-03-02

**Authors:** Ling Gu, Lin Li, Jinyang Sun, Yongqiao Zhao, Kai Wan, Meichun Zhang, Julin Li, Meihua Zhang, Guoding Zhu, Jianxia Tang

**Affiliations:** 1 School of Public Health, Nanjing Medical University, Nanjing 211166, China; glgl09@163.com (L.G.); lil1221@163.com (L.L.); sunshine55555@126.com (J.S.); 2 National Health Commission Key Laboratory of Parasitic Disease Control and Prevention, Jiangsu Provincial Key Laboratory on Parasite and Vector Control Technology, Jiangsu Provincial Medical Key Laboratory, Jiangsu Institute of Parasitic Diseases, Wuxi 214064, Chinaz02m14c588@aliyun.com (M.Z.); lijulin301@163.com (J.L.); zmh-07@163.com (M.Z.); 3 Wuxi School of Medicine, Jiangnan University, Wuxi 214122, China; wankai0613@163.com

**Keywords:** *Aedes albopictus*, microbiota, *Rahnella aquatilis*, mosquito reproduction

## Abstract

*Aedes albopictus* is one of the most important vectors of Dengue, which poses a serious threat to public health. The bacterial microbiota has an effect on the parameters of mosquitos, such as larval development and fecundity, and it has emerged as a promising field to be explored for novel environmentally friendly control strategies. *Rahnella* sp. are present in many insects, including *Ae. Albopictus*, and play a role in bacterial–insect interactions; however, the role of the bacteria in mosquito biology has not yet been characterized. In this study, we characterized the *Rahnella* isolate RAeA1 obtained from *Ae. albopcitus,* and its colonization stability in *Ae. albopictus* was investigated by generating GFP-tagged bacteria. The influences of the bacteria on larval development and mosquito reproductive capacity were evaluated by inoculating RAeA1 in axenic larvae and antibiotic-treated adult mosquitoes, respectively. The results indicated that RAeA1, which is widespread in the field population of *Ae. albopictus*, can be transmitted directly from the parental strain to the progeny and can rescue axenic larvae developing into adults with a prolonged development time to pupation. RAeA1 inoculation can impair egg production and ovary maturation, as well as reducing the synthesis of ecdysteroids and vitellogenin in *Ae. albopictus* females. Overall, our results provide a thorough study of bacterium function characterization that will facilitate the development of potential strategies in relation to the design of microbiomes for vector control.

## Simple Summary

*Aedes albopictus,* one of the most important vectors of Dengue, poses a serious threat to public health. The bacterial microbiota has an effect on the parameters of mosquitos; it is considered as a promising field for novel vector control strategies. *Rahnella* sp. are present in many insects including *Ae. albopictus*. Understanding the roles of bacteria in mosquito biology is crucial for developing effective control strategies. This study characterized the roles of the *Rahnella* isolate RAeA1 in development and egg production in *Ae. albopictus*. The RAeA1 isolate obtained from *Ae. albopictus* can be transmitted directly from the parental strain to the progeny and can rescue axenic larvae developing into adults with a prolonged development time to pupation. RAeA1 inoculation can impair egg production and ovary maturation, as well as reducing the synthesis of ecdysteroids and vitellogenin in *Ae. albopictus* females. This study provides a thorough exploration into bacterium function characterization in order to develop potential strategies in relation to the design of microbiomes for vector control.

## 1. Introduction

Mosquitoes are important vectors for a number of human pathogens, including arboviruses and protozoa, that pose a significant threat to public health. *Aedes albopictus* (*Ae. albopicuts*), the Asian tiger mosquito, is native to the tropic forests of Southeast Asia and has recently been reported as being common in Europe [1]; it is one of the 100 most invasive species in the world and is a highly efficient vector of numerous viruses that adversely affect human health, including Dengue, Zika, and chikungunya virus [2]. To date, chemical insecticides are still the most important measures for mosquito control. Due to the fact that most of these pathogens lack an effective vaccine and due to the increase in insecticide resistance in mosquitoes, the development and implementation of novel mosquito control strategies is necessary. The microbiome is involved in various aspects of mosquito life, such as the immune system, physiological and behavioral regulation, and metabolic response; it is becoming an important potential tool to deal with the aforementioned situation [3]. Understanding mosquito–microbiota relationships may have a great impact on the understanding of mosquito biology and on the development of innovative techniques for controlling mosquito-borne diseases, aiming to reduce mosquito vector capacity and/or inhibiting pathogen transmission [4]. Knowledge of the roles of microbes in the development, physiology, or immunity of their host is essential to reach this target.

The bacterial microbiota is known to heavily affect the health and fitness of mosquitoes [5,6,7], as well as influencing larvae growth, blood digestion, and immune function [5,8]. All mosquitoes are aquatic during their larval stages, and the majority of the microbiota is acquired through the water-based habitat in which the larvae develop [9,10]. Evidence has implied that there is a continuum of bacteria from the aquatic environment to both immature and adult mosquitoes, as shown by the overlap in bacterial composition between water, larvae, and adults [11,12,13]. Studies on *Anopheles* and *Aedes* larvae showed that a portion of their gut microbiota transmits to adults through metamorphosis [7]. In *Ae. albopictus*, some bacteria belong to the *Micrococcaceae*, *Pseudomonadaceae*, and *Staphylococcceae* families, are common to larvae and adults [14]. To date, most studies on microbiota in mosquitoes have been focused on the bacterial component, but the characterization of the symbiotic bacteria from mosquitoes and the role of these microbes in mosquitoes’ development and biology is still elusive, which will give an applied perspective for the control of mosquito-borne diseases.

*Rahnella* sp. is a Gram-negative bacterium from the family *Enterobacteriaceae*, which has been isolated from various environments including the water in which mosquito larvae live, the trees, human brain samples, and the gut of a beetle [15,16,17,18]. *Rahnella* sp. have also been isolated from many insects such as *Dendroctonus* and the western flower thrips, which played a role in the bacterial–insect interactions that contribute to their fitness, development, and survival [19,20]. Interestingly, *Rahnella* sp. is a microorganism that can be used as a biocontrol agent [21]. *Rahnella aquatilis* significantly reduced the incidence of diseases caused by *Botrytis. cinerea*, while *R. aquatilis* HX2 isolated from vineyard soils was shown to suppress grapevine crown gall disease caused by *Agrobacterium vitis* [22]. Including probiotics in honeybee nutrition, *Rahnella* sp. mitigates the toxic effects of clothianidin exposure on honeybee colony health [23]. Our previous studies indicated that *Rahnella* sp. is present in all developmental stages of *Ae. albopictus* (larvae, pupae, and adult mosquitoes), and was observed to be most abundant at the aquatic stages using Illumina Mi-seq next-generation sequencing through the V3 and V4 region of the bacterial 16S rRNA [24]. Identically, the genus *Rahnella* was present in *Ae. aegytpi*, *Ae. albopictus*, and *Culex* (*Cx*.) *quinquefasciatus*, both in those from the laboratory and those from the wild [25,26]. These findings suggested that *Rahnella* is a naturally occurring microorganism that can stably colonize in *Ae. albopictus* and may have important roles in mosquito biology, which has not yet been well characterized.

To gain insight into the potential roles of the *Rahnella* sp. in the physical function of *Ae. albopictus*, we characterized a strain of *Rahnella* (named RAeA1) that was present in different developmental stages of laboratory *Ae. albopictus* colonies, based on morphological, physiological, and phylogenetic analysis according to the 16S rRNA sequence. The distribution of the *Rahnella* isolate in the wild *Ae. albopictus* population was evaluated, and the vertical transmission ability was also investigated. Additionally, the role of the RAeA1 isolate on the growth of axenic larvae was assessed to evaluate the effects of the bacteria on *Ae. albopictus* development. Finally, we analyzed egg production, yolk deposition, ecdysteriod titers, and vitellogenin production to investigate the interaction of the bacteria with the female reproduction system.

## 2. Materials and Methods

### 2.1. Laboratory Ae. albopictus Maintenance

The lab-based *Ae. albopictus* colony was obtained in Jiangsu Province and has been maintained at the insectary at the Jiangsu Institute of Parasitic Disease (JIPD) since the 1980s; it was used for bacterial isolation in this study. Female mosquitoes were allowed to feed on sodium pentobarbital-anesthetized mice for 30 min to produce eggs. This process was reviewed and approved by the Institutional Ethics Committee of Jiangsu Institute of Parasitic Diseases: JIPD1-2022-004. The larvae were reared in plastic containers and fed with a mixture of pork liver powder and yeast powder. All *Ae. albopictus* adults were reared in screened cages and provided with 10% (*v*/*v*) glucose solution. The colony was routinely maintained at the insectary at 26 ± 1 °C and 70–80% relative humidity with a 12 h light/dark cycle.

### 2.2. Characterization of Rahnella sp. Isolate

*Rahnella* sp. named RAeA1, was the bacterium obtained from larvae, pupae, and adult mosquitoes (isolated and maintained in the National Health Commission Key Laboratory of Parasitic Disease Control and Prevention, Jiangsu Institute of Parasitic Diseases); it was used in this study. The total genomic DNA of the isolate was extracted using the DNeasy Blood and Tissue Kit (Qiagen, Hilden, Germany following the manufacturer’s instructions. The colony was passed three times to new agar plates to purify the isolate. Scanning electron microscopy was conducted to observe the cell morphology (Changsha Yanhao Testing Technology Co., Ltd., Changsha, China), as previously described [27]; the genomic DNA of the isolate was extracted for 16S rRNA amplification and sequencing. The 16S rRNA sequence and another thirteen 16S rRNA sequences from *Rehnella* sp. (obtained from the National Center for Biotechnology Information [NCBI] nucleotide database) were used for phylogenetic tree construction by applying the neighbor-joining method [28] in MEGA [29], which was visualized using iTOL [30]. The API (Analytical Profile index) 20E system (BioMérieux SA, Marcy l’Etoile, France) was used to identify the physiological features of the purified isolate, which was performed according to the manufacturer’s instructions. Briefly, the purified isolate was prepared with an overnight culture and inoculated in the strip with a bacterial suspension at 26 °C for 48 h; the color change in the tubes was observed and recorded according to the kit’s guide.

### 2.3. Quantitative PCR Amplification

Relative quantitative PCR was applied to evaluate the distribution of *Rahnella* sp. RAeA1 isolate in different developmental stages, organs of the laboratory colony, and the field population of *Ae. albopictus*. qPCR was constructed using a specific 16S rRNA gene segment (V3-V5 region) flanked by the RAeA1 isolation-specific PCR primers (RahF1: 5′-GAATATTGCACAATGGGCGC-3′; RahR1: 5′-GAGTTAGCCGGTGCTTCTTC-3′), and the mosquito rpS6 gene (rpS6F: 5′-GAAGTTGAACGTATCGTTTC-3′; rpS6R: 5′-GAGATGGTCAGCGCTGATTT-3′) was used as the reference gene [31]. The amplification was conducted in triplicate, each in a 10 μL reaction mixture containing 2 μL DNA template, 5 μL 2 × LightCycler 480 SYBR Green I Master (Roche, Basel, Switzerland), and 0.5 μM primer. Reactions were performed using the LightCycler 480 (Roche, Basel, Switzerland) with conditions of initial denaturation at 95 °C for 10 min, 45 cycles of 95 °C for 10 s, then at 60 °C for 15 s and 68 °C for 20 s for quantification; this was followed by a melting curve analysis of 95 °C for 5 s, 65 °C for 1 min, and 97 °C continuously held. The abundance of the RAeA1 isolate was calculated using the 2^−△Ct^ method.

### 2.4. Organ Dissection, Developmental Stages, and Field Ae. albopitus Collection

To evaluate the distribution of the RAeA1 isolate in different development stages of *Ae. albopicus*, 20 samples from the laboratory colony were pooled and collected in a corresponding 1.5 mL tube at each stage (L3 to L4 larvae and pupae; male and female with three biological replicates) and surface sterilized, and the DNA was extracted (DNeasy Blood and Tissue Kit, Qiagen, Hilden, Germany). The organs (ovary, fat body, salivary glands, Malpighian tubule, and midgut) from 120 laboratory-reared female adults (three to six biological replicates), aged 3 days, were dissected and used to analyze the abundance of RAeA1 isolate in different organs. After surface sterilization, organ dissection was performed in sterilized PBS. Dissected organs were then immediately pooled, and DNA was extracted (DNeasy Blood and Tissue Kit, Qiagen, Hilden, Germany).

To assess the distribution of the isolates in the field population, at least thirty samples were collected for L3 to L4 larvae, pupae (captured by dipping method), and adults (captured using a carbon dioxide baited trap) of *Ae. albopictus* from Hai’an, Tai’zhou, and Lian’yungang (three biological replicates) city in Jiangsu Province, China. The field-captured larvae and pupae were stored in plastic containers in their original habitat water, while adult mosquitoes were in plastic cages that were previously cleaned with 70% ethanol; after the capture, the samples were transported to JIPD for mosquito identification. Field-captured adults received no feeding (both glucose or blood feeding). Ten mosquitoes from each field were pooled for DNA extraction and qPCR quantification. The data were generated from three independent replications.

### 2.5. Generation of GFP-Tagged Bacteria

To verify the distribution and investigate the stable colonization ability of RAeA1, the green fluorescent protein gene eGFP was inserted into the chromosome of RAeA1; the targeting fragment (the upstream and downstream homologous recombination arms of the hupB gene of RAeA1 and the GFP-Apr sequence of pUC57_GFP-Apr) was cloned into pCVD442 to obtain the targeting plasmid pCVD442_hupB-GFP-Apr. It was transformed into the donor strain *E. coli* β 2155; the positive clones with GFP-Apr inserted and fused at the hupB gene position through bacterial conjunction were screened using PCR technology, and positive colons were named Rah/hupB-GFP-Apr. The primers used in the construction are listed in Table 1.

### 2.6. Introducing Bacteria to Ae. albopictus

Fifty newly emerged (3 days post-emergence) adult mosquitoes were supplemented with Rah/hupB-GFP-Apr to a final concentration of 10^9^ CFU/mL sterile 10% glucose solution. Three days later, the midgut, ovaries, and salivary glands of ten infected adult mosquitoes were dissected and photographed under a fluorescence microscope (BX63, Olympus, Tokyo, Japan). The remaining infected adult mosquitoes were fed on mice blood, and the eggs and larvae (2nd instar (L2) larvae) in sterilized water were collected and placed under a fluorescent microscope for imaging (BX63, Olympus, Tokyo, Japan).

### 2.7. Axenic (AX) and Conventional (CN) Culture of Ae. albopictus Larvae

For axenic (AX) larvae culture, the eggs from the general culture were surface-sterilized as previously described [32,33]. Briefly, the *Ae. albopictus* eggs were scraped from the oviposition paper onto a 0.2 μm filter membrane (Merck, Billerica, MA, USA) and then soaked in 70% ethanol for 5 min, followed by soaking in 1% bleach for 5 min, and finally incubated in 70% ethanol for 5 min. After rinsing with sterile water three times, the eggs were transferred to a disposable sterile culture dish containing sterile water for the first AX instars to be hatched. After hatching, the larva was placed into a sterilized 24-well plate with one larva in 2 mL of sterile water per well; a total of 50 μL of sterilized food was supplied to each well. The food was prepared by adding ground fish food (Tetra MinBaby, Herrenteich, Germany) (50 mg/mL) to sterile water and being autoclaved. The *Escherichia coli* (strain XL10, maintained in the National Health Commission Key Laboratory of Parasitic Disease Control and Prevention, Jiangsu Institute of Parasitic Diseases) and *Rahnella* sp. RAeA1 isolate were cultured in LB broth without antibiotics for 16 h; then, they were re-suspended in sterilized PBS to obtain a concentration of 10^9^ CFU/mL. An amount of 20 μL of aliquot was added to the RAeA1 (AX + RAeA1) and *E. coli* (AX + *E. coli*) group in AX larvae, respectively. Conventionally reared eggs without surface disinfection that were maintained in non-sterile water with fish food were used as controls. The culture dish was placed in a climate box with a relative humidity of 75%, a temperature of 26 °C, and a light/dark cycle of 12:12 h. About 120 larvae were observed for development metrics in each group.

### 2.8. Developmental Metrics

The development time to pupation (days) was assessed for both the conventional and AX groups, supplied with *Rahnella* sp. RAeA1 isolate or *E. coli*, respectively, with three different rearing cohorts consisting of at least 120 individuals per replicate. The eclosion rate was calculated by the adult number of both sexes divided by the total pupae number per replication times 100. The sex ratio was determined by counting the number of males and females separately and dividing it by the total number of adults per replication. The wing length of adults was used as a proxy for the body size of adults (measured by LAS X Life Science under the DVM6 Digital Microscope (Leica, Wetzlar, Germany)) [7,34]. Ten male and female individuals from different replicates were randomly selected to be measured.

### 2.9. Antibiotic Treatment and Bacteria Oral Feeding for Adult Mosquitoes

Conventionally reared adult mosquitoes were fed with 10% glucose containing penicillin–streptomycin (10 units/mL, 10 μg/mL, Solarbio, Beijing, China) continuously for 5 days, following previous studies [35,36]. Then, the mosquitoes were starved for 24 h to allow the antibiotics to metabolize prior to bacterial challenge. The removal of the microbes was confirmed using a colony-forming unit (CFU) assay [37]. After the antibiotic treatment, the mosquitoes were fed with sterilized 10% glucose solution in the antibiotic group, sterilized 10% glucose solution with 10^9^ CUF/mL RAeA1 in the antibiotic + RAeA1 group, and 10^9^ CUF/mL *E. coli* in the antibiotic + *E. coli* group for three consecutive days. The mosquitoes were then starved for 24 h and fed on mouse blood, as previously described. Mosquitoes from the same batch without antibiotic treatment and the same sterilized glucose solution or sterilized glucose solution supplied with the same bacteria as in the antibiotic groups were correspondingly applied as the control, RAeA1, and *E. coli* groups, respectively. One hundred 1-day-old female mosquitoes were included in each group, and the experiment was replicated twice.

### 2.10. Fecundity and Vitellogenesis Assay

The engorged female mosquitoes from 6 groups, as described above, were anesthetized with carbon dioxide and then placed singly in disposable cups at 24 h post blood meal (PBM). The cups were lined with wet filter paper to collect eggs. The number of eggs laid by a single mosquito was recorded for up to 7 days PBM and a total of 25–50 mosquitoes in each group were recorded. The length of the long diameter of each ovary [38] from 10 females was measured in μm.

The activation of the vitellogenic phase was assessed by measuring the ecdysteroid production of the ovaries, as well as the yolk deposition into oocytes and the vitellogenin production by the fat body. Yolk deposition into follicles was measured according to the ratio of yolk to follicle length in the follicles from 10 follicles in each pair of ovaries from the mosquitoes 24 h PBM; the ratio with the long diameter of follicles was calculated [39]. The edcysteriod titer was quantified using the 20-Hydroxyecdysone (20E) quantitative detection kit (Insect 20-Hydroxyecdysone ELISA Detection Kits, Enzyme-linked Biotechnology Co., Ltd. Shanghai, China) based on the enzyme-linked immunosorbent assay (ELISA), according to the manufacturer’s instructions. Briefly, according to the 20E standard, ovaries from each group were added to the microporous enzyme-labeled plate, which was pre-coated with anti-insect 20E antibody, and incubated with HRP-conjugate reagent. The optical density (O.D.) was read at 450 nm using a microtiter plate reader (Tecan spark, Mannedorf, Switzerland). The amount of edcysteroid in each group was calculated based on the standard curve generated according to the kit’s instructions. The ovaries were dissected at 6 h, 24 h, and 30 h PBM in each group; a total of 4 ovaries was used for each test, and 4 replicates were collected from each group. For vitellogenin detection, the remaining abdomens (without midguts, ovaries, heads, and Malpighian tubules) containing the fat body were collected 24 h PBM, and vitellogenin was detected using the Insect VTG ELISA Detection Kit (Enzyme-linked Biotechnology Co., Ltd., Shanghai, China) following the instructions provided by the company, which was identical to the ecdysteroid test. Four fat body measurements were collected in each test, and four replicates were performed in each group.

### 2.11. Statistics

All the graphs were generated using GraphPad Prism software version 10 (GraphPad Software Inc., San Diego, CA, USA). The data are expressed as the means ± SEMs. For categorical data (emergence rate, sex ratio), the Chi-square test (Fisher’s exact test) was applied. One-way and two-way ANOVA tests were used for comparison between multiple groups. A *p*-value below 0.05 was considered statistically significant.

## 3. Results

### 3.1. Morphological, Physiological, and Biochemical Characteristics of Rahnella sp. RAeA1 Isolation

**Figure 1:**
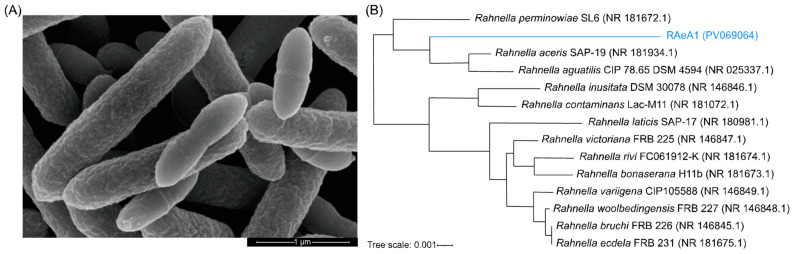
The microscopic and phylogenetic analysis of RAeA1 isolated from *Ae. albopictus*. (**A**) A scanning electron microscopic photo indicating group bacteria structure. (**B**) The phylogenetic tree of RAeA1. The tree was constructed using the neighbor-joining method without distance correction based on the 16S rRNA sequence using MEGA and was visualized using iTOL [30]. The 16S rRNA sequences of other *Rahnella* strains were obtained from the National Center for Biotechnology Information. Bootstrap support percentages (>50%) are shown at nodes and the bar represents 0.001 substitutions per nucleotide position.

One *Rahnella* sp. isolate, named RAeA1, was found to be Gram-reaction-negative, white-to-cream-colored, circular, and convex with an entire margin. The scanning electron microscope showed that RAeA1 represents short rods (0.9–1.0 × 1.3–1.7 μm), with capsules, without germ and flagella (Figure 1A). The nearly complete 16S rRNA gene sequence (1318 bp) (GenBank accession number: PV069064) of the RAeA1 isolate was independently determined and used to construct the phylogenetic tree using the neighbor-joining method. The NJ tree showed that the RAeA1 isolate forms a tight sub-cluster with *R. aquatilis* CIP 78.65 and *R. aceris* SAP-19 (Figure 1B), indicating that these two are closely related.

With regard to phenotype features, the RAeA1 isolate was subjected to 21 physiological and biochemical tests using the API 20E rapid bacterial identification kit. The strain was negative for ornithine decarboxylase, lysine decarboxylase, and arginine dihydrolase activities; however, it can produce acid from glucose, sucrose, lactose, mannitol, rhamnose, sorbitol, and arabinose, as well as having no hydrogen sulfide production (Table 2), which was consistent with a previous publication about *Rahnella* sp. [40]. The numbering in the API 20E Test Strip of the profile for this combination of reactions used the code 1004773, indicating that it is closely related to *R. aquatilis*. Based on the data presented here, the RAeA1 isolate was determined to be *R. aquatilis*.

### 3.2. Distribution of R. aquatilis RAeA1 Isolate in Developmental Stages, Tissues, and Field Populations of Ae. albopictus

**Figure 2:**
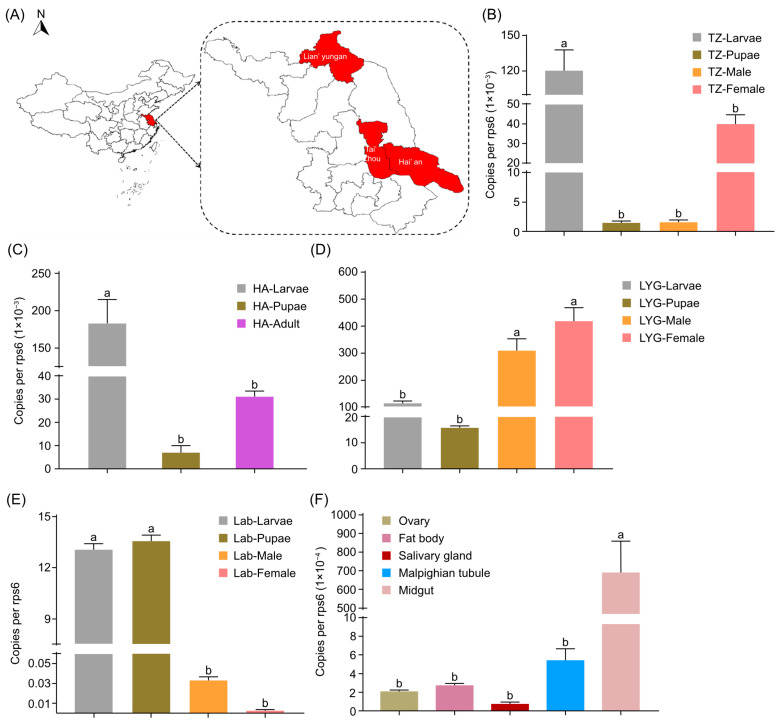
The location and distribution of the *R. quantilis* RAeA1 isolate at different developmental stages in field populations in Jiangsu Province (**A**–**D**), the laboratory (**E**), and different tissues (**F**). (**A**) The geographic location of Tai’zhou, Hai’an, and Lian’yungang in Jiangsu Province. Geographic information system (GIS)-based spatial analysis was conducted to demonstrate the study sites at a prefectural level in Jiangsu Province. All spatial analyses were carried out via QGIS (Quantum GIS, v2.10.1). (**B**) The infection of RAeA1 was observed using the relative qPCR method in different field-caught *A. albopictus* populations (F0 generations) from Tai’zhou (*F*_(3, 7)_ = 31.51, *p* = 0.0002), (**C**) Hai’an (*F*_(2, 6)_ = 25.35, *p* < 0.0001), (**D**) and Lian’yungang (*F*_(3, 8)_ = 29.38, *p* = 0.0001), Jiangsu, China. (**E**) The abundance of RAeA1 was determined using the relative qPCR method in different developmental stages from 3rd instar (L3) to 4th instar (L4) larvae, pupae, and male and female adults from the laboratory *A. albopictus* colony (*F*_(3, 8)_ = 3292, *p* < 0.0001). (**F**) The distribution of RAeA1 in different tissues (*F*_(4, 18)_ = 14.86, *p* < 0.0001) was also quantified using the same method. Values are means ± SEMs (error bar) of triplicate biological samples. Means that share the same letter are not significantly different and different letters indicate significant differences as determined by one-way ANOVA (with Holm-Šídák’s multiple comparisons test) analysis (*p* < 0.05). All the analysis was carried out using GraphPad Prism 10.

To determine how widespread the *R. aquatilis* RAeA1 isolate is, the abundance (bacterial density) of the RAeA1 isolate in the field was determined by collecting larvae (L3 to L4 instar), pupae, and adult mosquitoes, respectively, of *Ae. albopictus* from different areas of the Jiangsu Province and insectary. The relative qPCR results proved that the RAeA1 isolate existed in all the field population (Tai’zhou, Hai’an, Lian’yungang, and laboratory) (Figure 2A–E) we included in this study, indicating that the RAeA1 isolate is one of the common shared bacteria that exists in the *Ae. albopictus* population. The abundance of the RAeA1 isolate varies in the field and lab colony, with the highest density of larvae in Hai’an and Tai’zhou; the highest density of pupae in the laboratory; and the highest density of adults in Lian’yungang, respectively.

To determine the distribution characteristics of the *R. aquatilis* RAeA1 isolate in different tissues of *Ae. albopictus*, the midgut, ovary, fat body, Malpighian tubule, and salivary glands of mosquitoes maintained in the insectary were dissected, and relative qPCR analysis was performed. The results showed that RAeA1 can be detected in the Malpighian tubule, fat body, ovary, salivary glands, and midgut; it was most abundantly found in the midgut of *Ae. albopictus* (Figure 2F).

### 3.3. Distribution of Rah/hupB-GFP-Apr in Different Tissues and Transmission to Next Generation

**Figure 3:**
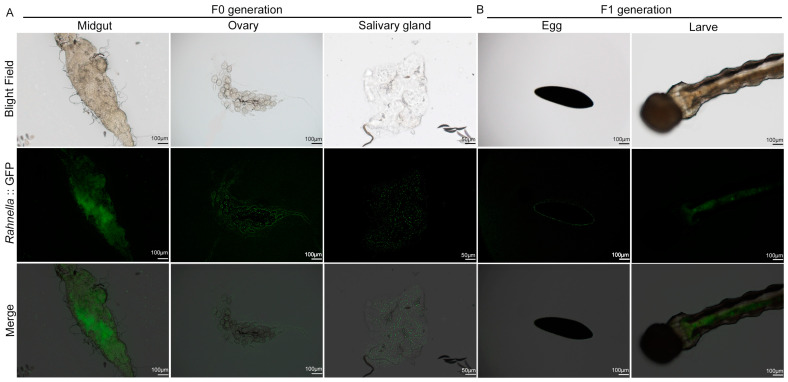
Distribution of Rah/hupB-GFP-Apr in various tissues of F0 generation and F1 generation *Ae. albopictus* (representative images). (**A**) One-day-old mosquitoes were fed Rah/hupB-GFP-Apr bacteria, and distribution of bacteria in midgut, ovary, and salivary glands was determined (scale bar: 100 μm or scale bar: 50 μm). (**B**) Distribution of Rah/hupB-GFP-Apr in eggs and larvae laid by female mosquitoes that were fed Rah/hupB-GFP-Apr bacteria for three days.

To verify the tissue distribution and evaluate stable colonization ability in the mosquitoes, the midgut and salivary glands were dissected and photographed under a fluorescence microscope after introducing Rah/hupB-GFP-Apr into the mosquitoes through oral feeding. The results showed the presence of fluorescent *R. quantilis* RAeA1 in the midgut, ovaries, and salivary glands (Figure 3A), which is consistent with the relative qPCR results. In addition, the eggs and second-instar larvae from Rah/hupB-GFP-Apr-fed mosquitoes after blood meal were collected in sterilized water and photographed. Luminescent bacteria can be detected around the eggs and larvae of the F1 generation (Figure 3B), which is consistent with qPCR results in the F1 generation from the field population (Supplementary Figure S1), indicating that RAeA1 can be stably present in the mosquito population through vertical transmission by female mosquitoes during oviposition.

### 3.4. Axenic Larvae Develop When Fed R. aquatilis RAeA1 Isolate but with Developmental Delay

**Figure 4:**
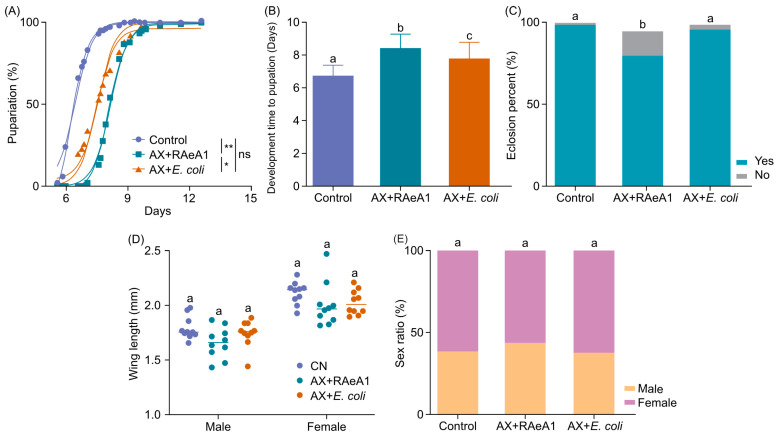
The development of axenic *Ae. albopictus* larvae supplied with RAeA1 and *E. coli*. (**A**) The percentage of axenic (AX) larvae that develop into pupae when supplied with RAeA1 (AX + RAeA1) and *E. coli* XL-10 (AX + *E. coli*) versus the percentage of conventional (CN) larvae that develop into adults over time (days). * *p* < 0.05; ** *p* < 0.01; ns: not significant. (**B**) Development times (days) from larvae to pupation in the CN, AX + RAeA1, and AX + *E. coli* groups. (**C**) The percentage of adults that emerged from the CN, AX + RAeA1, and AX + *E. coli* groups (Chi-square test and Fisher’s exact test). (**D**) Scatter plots showing the median wing length of adults (males and females) that emerge when supplied different bacteria. (**E**) The sex ratio of adult mosquitoes emerged from the groups when fed different bacteria (Chi-square test and Fisher’s exact test). Data are represented as means ± SEMs, and Student’s *t*-test was used in the analysis. Means that share the same letter are not significantly different, and different letters indicate significant differences (*p* < 0.05). All analysis was carried out using GraphPad Prism 10.

To assess whether the RAeA1 isolate can support the development of *Ae. albopictus*, we established the AX larvae, which were supplied with the *R. aquatilis* RAeA1 isolate or *Escherichia coli* (*E. coli*) strain XL10, which serves as a non-endogenous bacterial control, while the conventional cultured larvae served as the control. There were no axenic larvae that grew beyond the second instar in the present study, which has also been proven in previous studies [41,42]. The results showed that AX larvae supplied with RAeA1 (AX + RAeA1) or *E. coli* (AX + *E. coli*) can prevent the AX larvae from failing to grow into adults (Figure 4A); however, the developmental time (days) from larvae into pupation (mean AX + RAeA1 group: 8.4 days; AX + *E. coli* group: 7.8 days) was significantly delayed compared with the conventional culture conditions (control group: 6.7 days) (Figure 4B), suggesting that the full development of the larvae depends on the complete profile of the microbiota. In addition, the RAeA1 isolate cannot rescue the development of AX larvae as well as *E. coli,* with a longer development time to pupation (Figure 4B) and a lower eclosion percentage (Figure 4C). Supplementing RAeA1 or *E. coli* in AX larvae had no significant effects on the adult mosquitoes’ size, as estimated by the wing length (Figure 4D) and sex ratio (ratio of males to females in the control, AX + RAeA1, and AX + *E. coli* group was 0.61, 0.75, and 0.59, respectively) of newly emerged adults (Figure 4E). Taken together, these results suggest that both endogenous (RAeA1) and non-endogenous (*E. coli* XL-10) forms can rescue axenic larvae developing into adults, but with developmental delay.

### 3.5. R. aquatilis RAeA1 Isolate Impairs Egg Production and Ovary Maturation with Low Level of Ecdysteroids and Vitellogenin

**Figure 5:**
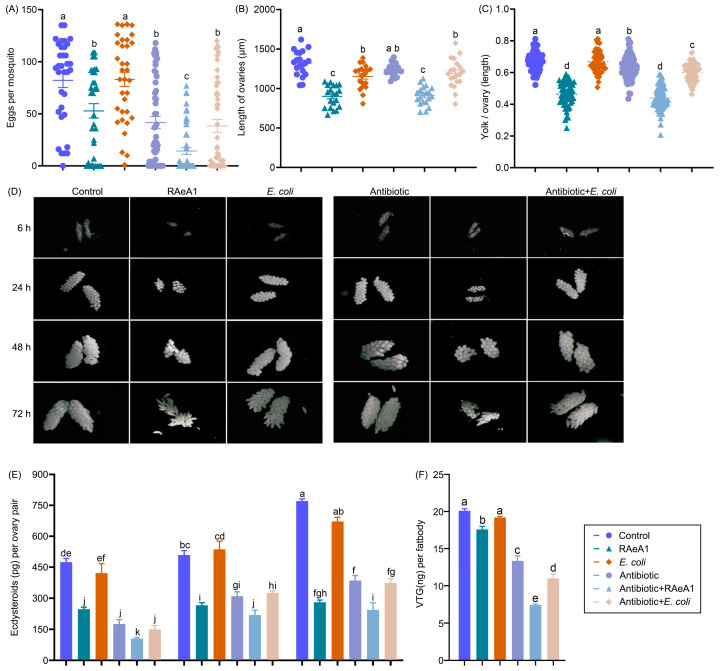
The egg production and ovary maturation of *Ae. albopictus* females supplied with RAeA1 and *E. coli*. (**A**) The total number of eggs laid per *Ae. albopictus* adult (*F*_(5, 246)_ = 19.91, *p* < 0.0001). Control: RAeA1 and *E. coli* (*n* = 34); antibiotic: antibiotic + RAeA1 and antibiotic +*E. coli* (*n* = 50). Control: conventionally reared; RAeA1: conventionally reared plus RAeA1; *E. coli*: conventionally reared plus *E. coli*; antibiotic: antibiotic treated; antibiotic + RAeA1: antibiotic treated before being fed with RAeA1; antibiotic + *E. coli*: antibiotic treated before being fed with *E. coli*. (**B**) Average ovary size (length of the long axis) isolated from randomly selected female mosquitoes in each group with different treatment (*n* = 20) after 24 h PBM (*F*_(5, 114)_ = 31.01, *p* < 0.0001). (**C**) The ratio of yolk to ovary by length 24 h PBM (*F*_(5, 401)_ = 154, *p* < 0.0001); control (*n* = 70), RAeA1 (*n* = 56), *E. coli* (*n* = 54), antibiotic (*n* = 76), antibiotic + RAeA1 (*n* = 75), and antibiotic + *E. coli* (*n* = 90). (**D**) Representative images of ovaries dissected from conventionally reared, RAeA1-inoculated, *E. coli*-infected, antibiotic-treated, antibiotic treatment before RAeA1 infection, or *E. coli*-infected female mosquitoes at 6 h, 24 h, 48 h, and 72 h PBM, respectively. All images were taken with LAS X Life Science under the DVM6 Digital Microscope (scale bar: 100 μm). (**E**) Ecdysteroids produced per ovary by females in each group at 6 h, 24 h, and 30 h PBM. A two-way ANOVA (with Holm-Šídák’s multiple comparisons test) was used in the analysis (comparing cell means regardless of rows and columns with a row factor (time) of *F*_(2, 202)_ = 87.47, *p* < 0.0001; column factor (groups): *F*_(5, 202)_ = 102.7, *p* < 0.0001). (**F**) Vitellogenin produced per fat body by females in each group (*F*_(5, 36)_ = 149.6, *p* < 0.0001). In (**A**–**C**,**F**), one-way ANOVA (with Holm-Šídák’s multiple comparisons test) was applied in the analysis. All data are presented as means ± SEMs. Means that share the same letter are not significantly different, and different letters indicate significant differences (*p* < 0.05). All analysis was carried out using GraphPad Prism 10.

By reducing the number of mosquito eggs laid, the quantity of mosquito offspring can be effectively reduced, thereby reducing the chance of disease transmission. To investigate the influence of RAeA1 in egg production, we inoculated RAeA1 or *E. coli* to either conventionally reared or antibiotic-treated females; the removal of the microbes was confirmed using a colony-forming unit (CFU) assay (Supplementary Figure S2). The number of eggs laid by a single mosquito was recorded. The results showed that both females with and without antibiotic treatment laid smaller egg clutches (52.79 ± 6.94 /female in the RAeA1 group and 14.2 ± 3.27/female in the antibiotic + RAeA1 group) post-RAeA1 inoculation, compared to those inoculated with *E. coli* (83.06 ± 6.84/female in the *E. coli* group and 38.34 ± 6.13/female in the antibiotic + *E. coli* group) or glucose only (82.12 ± 6.89/female in the control group and 41.58 ± 5.79/female in the antibiotic group) (Figure 5A), which may not be due to the antibiotic treatment and bacteria inoculation (Supplementary Figure S3). To explore whether the reduced egg number produced by females supplied with RAeA1 was due to the defects of ovarian development, the length of the ovaries was measured at 24 h PBM. The results indicating that the sizes of ovaries (PBM) were also significantly smaller in groups when females were fed with RAeA1 (Figure 5B). In addition, yolk packing into the oocytes of follicles represented by the yolk/ovaries length ratio after 24 h PBM, which is a measure of egg maturation, were also analyzed in this study. The results also showed that follicles packed less yolk when females were inoculated with RAeA1 in both conventionally reared and antibiotic-treated mosquitoes compared to their counterpart groups, respectively (Figure 5C). The development process of ovaries from each group was continuously observed from 6 h to 72 h PBM, which also proved that the ovaries from the RAeA1-infected females showed a delay of ovary development and with a lower number of follicles (Figure 5D). All these data suggested that RAeA1 infection in female mosquitoes may suppress ovary development. We did not rear these eggs to larvae to assess whether differences existed in the subsequent hatching, growth of larvae, or pupation and adult emergence.

To further investigate the underlying mechanisms for RAeA1-induced defects in ovary development post blood meal, ecdysteriods—which are the key hormones governing female mosquito reproduction—and vitellogenin, the yolk protein biosynthesized by fat body in response to ecdysteriods, were analyzed in this study. The level of ecdysteriods in the ovaries was detected at 6 h, 24 h, and 30 h PBM, and vitellogenin in fat body was measured at 24 h PBM. The results revealed that the ovaries produced less ecdysteriods at different time points (6 h, 24 h, and 30 h PBM) in the present study (Figure 5E), while the fat body also produced less vitellogenin (Figure 5F) in females inoculated with RAeA1 in both the conventionally reared group and the RAeA1 and the antibiotic treatment group before being supplied with RAeA1. Collectively, these results strongly suggest that *R. aquatilis* RAeA1 isolation impaired the egg production and ovary maturation of *Ae. albopictus* by reducing ecdysteriod production in the ovaries and vitellogenin synthesis in the fat body.

## 4. Discussion

The *Rahnella* RAeA1 isolate, obtained from lab-reared *Ae. albopcitus*, was characterized through morphology (Figure 1A), 16S rRNA sequence analysis (Figure 1B), and physiological methods (Table 1); it was proven to be *R. aquatilis*. The abundance of the *Rahnella* RAeA1 isolate in different developmental stages from the lab-reared and field-caught populations of *Ae. albopictus* mosquitos (Figure 2A) was evaluated; the results showed that the RAeA1 isolate was most abundant at the larvae stage in the Tai’zhou (Figure 2B) and Hai’an (Figure 2C) field populations, in adult females in the Lian’yungang field population (Figure 2D), and at the pupae stage in the lab colony mosquitoes (Figure 2E). The bacteria abundance differs between mosquitoes, which may be a function of collection location; adult mosquitos collected were not from the same batch of larvae and pupae in the field, which may also contribute to this difference. Together, these results suggest that *Rahnella* is one of the widespread bacteria in *Aedes* mosquitoes. Like many other constituent microbes which have established themselves as commensal, *Rahnella* sp. may have important roles in the vector life cycle [43,44,45], which lays the foundation for further research on the impact of *Rahnella* on the development of *Ae. albopictus*.

In addition, to date, most studies that examine the bacterial composition of tissues have focused on the adult gut. However, the mosquito bacterial microbiota is not exclusive to the gut, and has been described in other tissues, such as the salivary glands [13] and reproductive tract [46]. The RAeA1 isolate can also be detected in the salivary glands and the ovaries, as well as the midgut (Figure 2F), as in our study, suggesting that it may also have an influence on productive parameters in *Ae. albopictus*. By introducing fluorescent plasmids into an RAeA1 isolate to obtain Rah/hupB-GFP-Apr, we demonstrated that RAeA1 can also be detected in the midgut, salivary glands, and ovaries (Figure 3A), which is consistent with qPCR results. It can also be vertically transmitted to *Ae. albopictus* (Figure 3B), which is of great significance for RAeA1 in order for it to become one of the alternative microorganisms for biological control.

We then further systematically investigated the function of RAeA1 in the larvae development and reproduction of female *Ae. albopictus.* The results in relation to larvae development showed that the *R. aqualitis* RAeA1 isolate and *E. coli* can both rescue the AX larvae from failing to grow into adults (Figure 4A,B), which is consistent with previous studies, as several different bacterial species rescued the development of gnotobiotic larvae [12]. In previous studies, the pupation time of *Ae. aegypti* larvae exposed to *Rahnella* was longer than that of the control group or larvae that were exposed to a single bacterium such as *Revsonella*, *Flexibacterium*, or *Paenibacillus* [47]. Similarly, in our study, the RAeA1 isolate cannot rescue the development time to pupation as well as *E. coli* with a prolonged development from larvae to pupae; further studies are required to decipher the exact mechanism(s) causing this phenotype.

Previous studies have found that bacteria are one of the indispensable conditions for female mosquitoes to lay eggs. In the absence of intestinal microorganisms, mosquitoes lay fewer eggs [7,39]. From a vector control standpoint, reducing egg production will greatly influence the population size and pathogen transmission of vector mosquitoes. In the present study, the influence of the RAeA1 isolate on the reproduction of *Ae. albopictus* was investigated; we supplement the RAeA1 isolate or *E. coli* to either conventionally reared or antibiotic-treated females to evaluate the egg production, ovary development (measured by ovary length), and yolk packing into oocytes. Our results show that treating either conventionally reared or antibiotic-treated females inoculated with the RAeA1 isolate resulted in reduced egg numbers (Figure 5A) and sizes of ovaries (Figure 5B), suggesting that RAeA1 infection impairs egg production. Treatment with antibiotics reduced the abundance of cultivable gut bacteria, resulting in the slower digestion of the blood bolus and significantly reducing the number of laid eggs [48]. Our results also proved that the females in the antibiotic-treated group laid less eggs than in conventionally reared females (Figure 5A), with less yolk packing in follicles (Figure 5C).

From earlier studies, some symbiotic bacteria can mediate the uptake of vitellogenin by oocytes to promote egg development [49], while some bacteria such as *Wolbachia* inhibit the ovary development of mosquitos [50]. The smaller egg clutches laid by females inoculated with the RAeA1 isolate either in the conventional or antibiotic-treated groups are associated with defects in yolk packing in oocytes (Figure 5C), with a delay of ovary development and lower follicle numbers (Figure 5D), indicating that the RAeA1 isolate may have negative effects on the reproduction of female mosquitoes. One of the main hormones regulating reproduction in adult Diptera is ecdysteroids. After blood meal, the head of adult mosquitoes secretes the ovarian ecdysteroidogenic hormone (OEH); then, the ovaries synthesize and secrete ecdysteroids under the stimulation of OEH. Ecdysteroids become the activated form of 20-hydroxyecdysone (20E) in the fat body [51]. 20E is one of the key regulatory hormones that promotes vitellogenesis and activates vitellogenin (Vg) gene expression in the fat body of female mosquitoes; it subsequently synthesizes vitellogenin (VTG) [52], which is taken up by the ovaries and converted into vitellin, thus providing the necessary nutrients, such as fat and protein, for egg development [53]. Our measures of ecdysteroids and vitellogenin levels in the ovaries and fat body, respectively, revealed that the average level of ecdysteroids in the ovaries and vitellogenin in fat body was lower in *Ae. albopictus* infected with the RAeA1 isolate compared with their counterpart groups, respectively. These results indicate that the RAeA1 isolate impaired the reproduction of females by reducing the level of ecdysteroids and vitellogenin.

This study has its limitations. Firstly, antibiotic treatments are the main approach used to manipulate mosquito bacteria communities for the purpose of investigating the functional roles of insect microbiota. However, this method may fail to eliminate all the bacteria (a colony-forming unit (CFU) assay was performed with negative results—Supplementary Figure S2), which may cause the bias of the results. Secondly, we fed mosquitoes in the reproduction experiment with living anesthetized mice, which may cause the mosquitoes to acquire microbes from the skin of the mice through blood-feeding; thus, the antibiotic-treated mosquitoes likely ceased to be gnotobiotic. In addition, we only investigated the role of RAeA1 in mosquitoes from a lab-reared colony of *Ae. Albopictus*; further studies may need to investigate field populations. Finally, only the number of eggs in treated mosquitoes was assessed; to verify the viability of the eggs, this also needs to be carried out in further experiments.

## 5. Conclusions

The results in this study show that *Rahnella* sp. RAeA1 is widespread in field populations and commonly exists in all developmental stages in *Ae. albopictus*. Our results further indicate that the RAeA1 isolate can stably colonize in *Ae. albopictus* generations and can rescue axenic larvae developing into adults, but with a prolonged development time to pupation. In addition, the *R. aquatilis* RAeA1 isolate impairs egg production and ovary maturation through a reduction in the synthesis of ecdysteroids and vitellogenin in *Ae. albopictus* females. Taken together, our study systematically investigated the role of specific symbiotic bacteria in development and egg production in the anautogenous mosquito *Ae. albopictus*, providing a basic understanding of microbe–mosquito interactions in order to develop strategies to create mosquitoes and design microbiomes that induce desirable properties for vector control.

## Figures and Tables

**Table 1:** Primers used in the construction and identification of GFP-tagged RAeA1 bacteria.

Name	Sequence (5′–3′)	Purpose
GFP-5F	GTATCGCTATGTGCACTGCTTTGGTGTC	Upstream homologous arm
GFP-5R	GTTTACGGCGTCTTTCAACGCTTTTCCTG	
GFP-3F	TTACAGCGTCGAAACGATTAGCAACAC	Downstream homologous arm
GFP-3R	CTTTCAGCTCATCGTCTGTAGCGGTC	
GFPApr-F	CAGGAAAAGCGTTGAAAGACGCCGTAAACCGTGGTTCTGGTGGTGAAGCAG	GFP-APR amplification
GFPApr-R	GTGTTGCTAATCGTTTCGACGCTGTAAGGAATAGGAACTTATGAGCTCAGCCAATC	
GFP-outF	GTGCTGAGAAGCTGGGCATTAACG	Outer identification
GFP-outR	CACTTTGTCTTGTAGTGAGGTAGCATCCAG	
GFP-R	CCATTAGTTGCATCACCTTCACCTTCAC	Insertion identification
Apr-F	GGCAGAGCAGATCATCTCTGATCCATTG	
GSF	GTTCGTGCCTTCATCCGTTTC	Sequencing
442R	AGAAGCCCTTAGAGCCTCTCAAAG	Sequencing
GFP-seqF	GGTTCTTTCACCGTGCGTGAG	Sequencing

**Table 2:** Physiological and biochemical characteristics of RAeA1 isolate.

Characteristic		RAeA1
	Gelatin hydrolysis	−
	Hydrogen sulfide production	−
	N_2_	+
	NO_2_	+
Acid production from	D-Glucose	+
	D-Mannitol	+
	Lactose	+
	D-Srobitol	+
	Inositol	−
	L-rhamnose	+
	D-saccharose	+
	D-melbiose	−
	Amygdalin	+
	L-arabinose	−
	Citrate	−
Enzyme activities	Arginine dihydrolase	−
	Lysine decarboxylase	−
	Orinithine decarboxylase	−
	Phenylalanine deaminase	−
	Urease	−
	Tryptophanase	−

+: positive; −: negative.

## Data Availability

All data generated or analyzed during this study are available in the article and Supplementary Materials.

## Supplementary Materials

The following supporting information can be downloaded at https://www.mdpi.com/article/10.3390/insects16030257/s1. Figure S1: Distribution of *R. quantilis* RAeA1 isolate at different developmental stages in Lian’yungang F1 generation, Jiangsu, China. Figure S2: Colony forming unit assay verifying antibiotic treatment. Figure S3: The percentage of blood-fed female *Ae. albopcitus* mosquito supplied with RAeA1 and *E. coli*.

## Abbreviations

LB: Luria–Bertani; CFU: colony-forming unit; AX: axenic; CN: conventional; PBM: post blood meal; L3: 3rd instar; L4: 4th instar; 20E: 20-Hydroxyecdysone; VTG: vitellogenin; OEH: ovary ecdysteroidogenic hormone.
