# Supplementary material for: Rahnella aquatilis Isolated from Aedes albopictus Impairs Mosquito Reproduction Capacity
Source: Insects. 2025 Mar 2;16(3):257. doi: 10.3390/insects16030257 (PMC11942639; doi:10.3390/insects16030257)
Supplement: Supplementary file 1 [file insects-16-00257-s001.zip › insects-3467010-supplementary.pdf]

Vertical transmission ability of RAeA1 isolate

Adult *Aedes albopictus* mosquitoes were collected from Lian'yungang and fed with mouse blood for 30 minutes. Two days later, the eggs were collected using UV-sterilized plastic dishes filled with sterile water. We collected eggs (3 tubes, 30 eggs in each tube) and rinsed the eggs 3 times with sterile water, soaked them in 70% alcohol for 5 min, and finally rinsed with sterile water, the eggs were then for qPCR detection. The remaining eggs were raised under conventional conditions afterwards. The larvae, pupae, male and female adult mosquitoes were collected in sequence during the growth of mosquitoes (3 tubes were collected from each group, with ten individuals in each tube), and the samples were then treated as described above. DNA was extracted and qPCR was carried out for the bacteria detection. The results indicated that RAeA1 can be detected in all development stages including eggs (Figure S1).

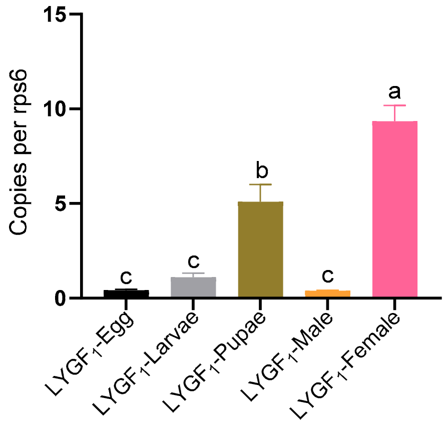

Figure S1. Distribution of *R. quantilis* RAeA1 isolate at different developmental stages in Lian'yungang F1 generation, Jiangsu China ($F_{(4, 10)} = 144.5$, $P = 0.0001$). Means which share the same letter are not significantly different and different letters indicate significant differences as determined by one-way ANOVA (with Holm-Šídák's multiple comparisons test) analysis ($P < 0.05$). The analysis was carried out by GraphPad Prism 10.

Generation of antibiotic-treated mosquitoes and verification

Mosquitoes were provided with cotton soaked with a 10% glucose solution including penicillin-streptomycin (10 units/mL, 10 μg/mL, Solarbio, Beijing, China) for 5 days to remove gut bacteria. The mosquitoes were then starved for 24 hrs. Three female mosquitoes were randomly selected, soaked in 70% ethanol for 3 minutes, washed with PBS for 3 times, and then added with 1 mL PBS to grind the mosquitoes. The obtained

suspension was diluted to 10^{-5} with 0.8%Nacl at a gradient of 10 times, and 200 μL dilution was smeared (LB medium plate without Ampiciline) and repeated for 3 times. The plate was cultured in an incubator at 26°C for 48-72 hours. Removal of gut bacteria was confirmed by a colony forming unit assay (Figure S2).

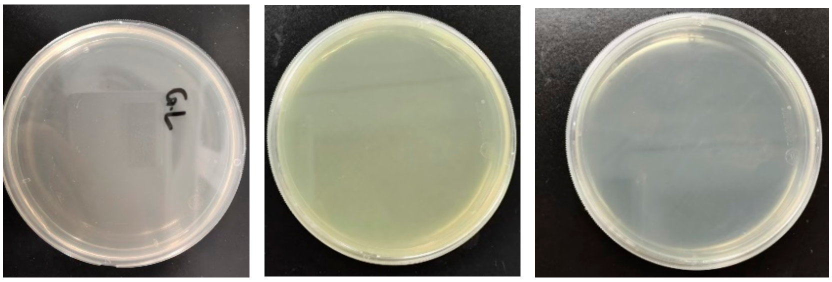

Figure S2. Colony forming unit (CFU) assay verifying antibiotic treatment (From three individual mosquito)

The effect of antibiotic treatment and bacteria oral feeding on blood feeding ability of female mosquitoes

Adult mosquitoes from conventional reared were treated by feeding mosquitoes with 10% glucose containing penicillin-streptomycin (10 units/mL, 10 μg/mL, Solarbio, Beijing, China) continuously for 5 days. Then, the mosquitoes were starved for 24 hours to allow the antibiotics metabolized to bacterial challenge. After the antibiotic treatment, the mosquitoes were fed 10% glucose solution in Antibiotic group, sterilized 10% glucose solution with 10^9 CUF / mL RAeA1 in Antibiotic + RAeA1 group, and with 10^9 CUF / mL *E. coli* in Antibiotic + *E. coli* group for consecutive three days (one hundred female mosquitoes were included in each group). The mosquitoes were then starved for 24 h and fed on mice blood and the number of blood-fed mosquitoes was recorded afterwards. The results showed that there was no statistically difference between each groups (Figure S3), indicating that antibiotic treatment and bacteria inoculation do not affect the blood feed desire of female mosquitoes.

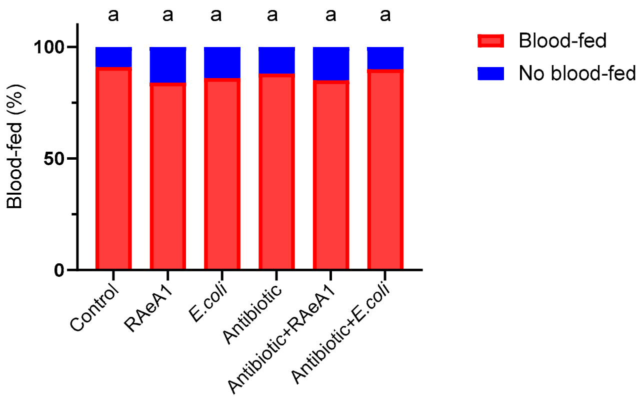

Figure S3: The percentage of blood-fed female *Ae. albopictus* mosquito supplied with RAeA1 and *E. coli*. Means which share the same letter are not significantly different (Chi-square test - Fisher's exact test).
